# ZnO Nano-swirlings for Azo Dye AR183 photocatalytic degradation and antimycotic activity

**DOI:** 10.1038/s41598-022-17924-3

**Published:** 2022-08-18

**Authors:** Khalid Umar, Manar Fawzi Bani Mfarrej, Qazi Inamur Rahman, Mohammad Zuhaib, Amir Khan, Qamar Zia, Saeed Banawas, Hera Nadeem, Mohd. Farhan Khan, Faheem Ahmad

**Affiliations:** 1 School of Chemical Sciences, Universiti Sains Malaysia, 11800 Gelugor, Pulau Pinang Malaysia; 2 Department of Life and Environmental Sciences, College of Natural and Health Sciences, Zayed University, 144534 Abu Dhabi, United Arab Emirates; 3 Department of Chemistry, Integral University, Lucknow, 226026 India; 4 Department of Agriculture, School of Sciences, Noida International University, Gautambuddha Nagar, 203201 India; 5 Department of Botany, Aligarh Muslim University, Aligarh, 202002 India; 6 Health and Basic Science Research Centre, Majmaah University, Al-Majmaah, 11952 Saudi Arabia; 7 Department of Medical Laboratory Sciences, College of Applied Medical Sciences, Majmaah University, Al-Majmaah, 11952 Saudi Arabia; 8 Department of Biomedical Sciences, Oregon State University, Corvallis, OR 97331 USA; 9 Department of Science, Gagan College of Management and Technology, Aligarh, 202002 India

**Keywords:** Biological techniques, Biotechnology

## Abstract

The sol-gel technique was used to fabricate ZnO Nano-swirlings (ZNsw) at a predetermined agitation rate (of >> 1900 rpm), with around 21.94 gm of zinc acetate dihydrate and 0.2 g cetyltrimethylammoniumbromide (CTAB) and a cationic surfactant (drop-wise). The impact of the predetermined agitation condition on the molecular size and morphology of ZNsw is examined, and the outcomes are dissected by useful characterization tools and techniques viz. XRD, SEM embedded with EDS, TEM, FT-IR and UV–visible. The SEM and TEM results suggest that the product formed into a big cluster of adequate ZNsw, containing a significant quantity of folded long thread-lengths. Each group indicated a fair amount of the volume of these lengths. The photocatalytic process of ZNsw was carried out as a result of the irradiation time due to the deterioration of Azo Dye AR183, resulting in approximately 79 percent dye discoloration following an 80-min UV light irradiation in the presence of ZNsw. Additionally, the synthesized ZNsw was tested for antagonistic activity, and the growth hindrance of two plant pathogenic fungal strains found. Per cent inhibition in growth of *Rhizoctonia solani *and *Alternaria alternata *were observed in response to ZNsw.

## Introduction

Zinc oxide with formula ZnO is a semiconductor of type- ‘n’, belonging to the group II-IV with a bandgap energy of 3.3 eV and high binding energy of 60 meV on 298 K^1^. ZnO has many outstanding properties, such as better clarity, strong electron susceptibility, and heavy blue-green luminosity at ambient temperature and UV absorption, making it ideal for the protection of the sunlight, textiles, catalysts, philters, photo-detectors, and the acquisition of solar energy^2–4^. Different forms of toxic substances originating from drugs, tinctures, surfactants, pharmaceuticals, and many other chemical industries, such as herbicides, fungicides, insecticides, etc. all of which can contribute to significant water contamination, soil and air pollution^5,6^. In the interests of humans, flora as well as of fauna, it is also imperative that all dangerous and poisonous contaminants entering different bodies of water should be regulated and that good steps are taken for purification of the numerous water supplies that are used to enhance life. This has been shown in numerous experiments with various contaminants such as herbicides, fungicides, insecticides, various pesticides and different drugs, dyes as well as surfactants that can be fully mineralized in the presence of different nanostructures of ZnO^7,8^. In different nanoscale ZnO synthesis, the different stirring conditions usually involve persistent executions before vigorous using it vigorously; even small temperatures to elevated temperatures, lasting from a few seconds to a many days, etc^9^. Madathil^10 ^synthesized ZnO-NPs by preparing stock solutions of zinc acetate in methanol and adding sodium hydroxide with nonstop stirring. Khan^11^ also synthesized ZnO-NPs in the presence of zinc acetate, ethylene glycol, and glycerol at 150 °C followed by mixing of 2-propyl alcohol and later triethylamine and then further prolonged heating. Zhang^9^ synthesized ZnO-NPs by continuously swirling zinc nitrate, bamboo pulp and Multi-amide compound for 40 min. Selim^12^ carried out biogenic synthesis of ZnO-NPs by heating crude plant extract of *Deverratortuosa*on magnetic stirrer and then added zinc nitrate hexahydrate till appearance of white precipitate.

As multifarious NPs have been exploited in the pollutant removals from aqueous solutions, previously various studies were moved forth for showing the efficiency of ZnO-NPs for photo-degradation of different dyes, organic pollutants etc^13–15^. Jayappa^16^ produced ZnO-NPs and observed methylene blue dye removal by three different samples of ZnO-NPs, namely L-ZnO-NPs, S-ZnO-NPs, and C-ZnO-NPs and discovered that in the presence of UV radiation, around 30% of L-ZnONPs, 30% of S-ZnO-NPs, and 90% of C-ZnO-NPs of methylene blue dye were deteriorated after 100 min; 100 min, and 120 min., respectively. Shrivastava prepared ZnO and 2%Fe–ZnO nanomaterials by using a low-cost sol–gel method and observed maximum degradation of methylene blue dye was 86% (ZnO) and that of 92% (2%Fe–ZnO)^17^. With other nanomaterials viz. SnO_2_ nanorods and NiO nanoparticles, Shrivastava observed maximum degradation of methylene blue dye as 94% and 98.7% resepectively^18,19^. Talebian^20^ synthesized different morphologies of ZnO-NPs using simple solvothermal process with different solvents and the photo-degradation of acid orange 74 (CI 18745), an Azo Dye, were tested. Uribe-López^21^ prepared ZnO-NPs by two routes and designated as ZnO-PP (for precipitation method) as well as ZnO-SG (for sol–gel method) and noted the effect of the synthesis method in the photocatalytic efficacy of ZnO-NPs for the degrading and mineralizing a refractory pollutant, phenol. Kim^22^ tested the effectiveness of Ag-NPs on ascomycetous fungi that cause oak wilt under in vitro. An easy synthesis of ZnO Nano-swirlings (ZNsw) using the sol–gel process in the presence of dihydrate zinc acetate under a prescribed agitation setting (>> 1900 rpm) and in the surrounding of cationic surfactant, CTAB; sodium hydroxide has been added. The impact of specified agitation parameter on particle size as well as on morphology of the synthesized ZNsw lead to explore a simple production mechanism. The predetermined agitation rate (of >> 1900 rpm) is a significant criterion in customising the architecture of nanoparticles, which is further sparked to geometrical of facet i.e. < 0 0 0 I > whenever the agitation rate varies, for alteration in aspect ratios (L/D fractions)^23–26^. In addition, several techniques such as XRD, SEM with EDS, TEM, FTIR, and UV–vis spectroscopy were utilized to characterize ZNsw. Here, we further analyze the competence of the photocatalytic behaviour of the synthesized ZNsw, under a source of ultraviolet light by observing the deterioration of Azo Dye AR183 as a function of the time of irradiation. The two fungal pathogens viz., *R. solani* and *A. alternata* causes sheath blight in rice and leaf spot disease in *Withania somnifera* (Ashwagandha) respectively. Plant diseases are the major constraint in the yield production of plants. It is challenging to control fungal pathogensresisttraditional fungicides such as benzimidazoles and dicarboximides. To solve this resistance problem, it is necessary to exploit the potential of novel antifungal agents, which may replace traditional plant disease control strategies. In recent years, NPs have gained popularity due to their distinct physical and chemical properties which differ significantly from their conventional counterparts. Recently, scientists have shown keen interest in studying the antimicrobial activities of various NPs materials, including silver, copper, titanium dioxide, and zinc oxide.

Moreover, among various types of dyes, Azo Dyes represent the largest class which are categorised as the organic pollutants and extensively associated with various essential sectors such as textile industries, food industries and printing as well as cosmetic manufacturing industries^27^*.* Furthermore, Azo Dye AR183, represents the class of reactive dyes which shows highly carcinogenic nature and recalcitrant to degradation^28^ Therefore, in addition to the possible use for the deterioration of Azo Dye AR183, ZNsw has also been tested for fungicidal activity against two fungal strains (*R. solani* and *A. alternata*), to use ZNsw as an appropriate fungicide for agriculture and food protection.

## Results and discussion

### Morphological nature, structural properties and optical characteristics of synthesized ZNsw

**Figure 1:**
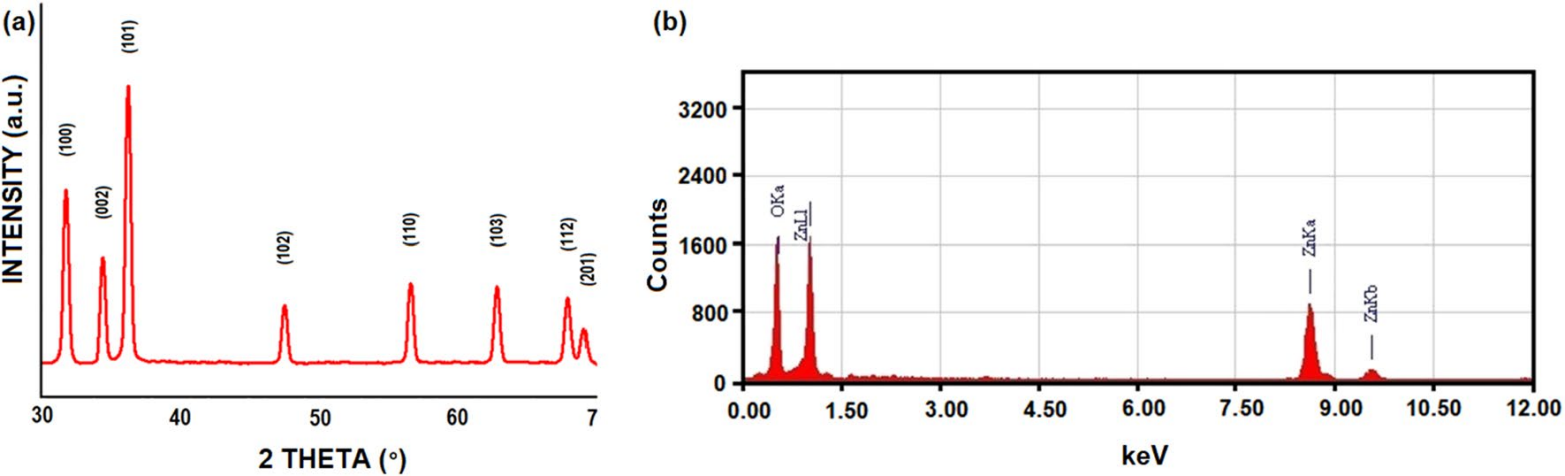
(**a**) X-ray diffraction pattern and (**b**) EDX as synthesized ZNsw via sol–gel process.

The synthesized ZNsw crystallinity as well as phase of the crystal of ZNsw samples, was calculated by X-ray diffraction and shown in Fig. 1a. As reported by ICDD data (Card Number: 080-0075), all diffracted peaks completely correlated to the regular peaks. The hexagonal wurtzite structure typical for the ZNsw samples, where a, b = 3.24 Å & c = 5.20 Å; where a/c = 0.62, was illustrated. The XRD pattern then revealed that the ZNsw samples were developed in a single phase. The form of the peaks depicts the smaller magnitude and the samples crystalline nature. There are no peaks other than the regular peaks shows that the ZNsw synthesized is free of some sort of impurity. The strongest peak was collected along the (101) alignment at 2θ = 36.2°^29^. The average sizes of the particles obtained when fabricating ZnO Nano-swirlings (ZNsw) at a predetermined agitation rate (of >> 1900 rpm) was estimated to be around ∼16 and ∼48 nm, respectively, when utilising the Debye Scherrer equation (from dominant peak depending on the full width at half maximum (β), FWHM), implying that peak widths and crystal sizes are inversely attributed.

Average crystalline sizes were compared using the Debye–Scherrer Eq. (1) and Williamson–Hall Eq. (2), as follows:

1 $$D = \frac{k\lambda }{{\beta \cos \;{\uptheta }}}$$

2 $$\beta \cos \, \theta = 4\varepsilon \sin \, \theta + \frac{k\lambda }{D}$$

where *D* Crystallite size (nm); *k* Constant (0.89 for heterogeneous particles); *λ* Wavelength of X-rays (Cu-Kᾳ = 0.1541 nm); *β* full width at half maximum, FWHM (of broad & intense peaks); *θ* Bragg’s angle.

The interaction of capping agents and precipitating agents in a pre-defined agitation condition at particularly high pH, results in the formation of ZNsw crystals. Due to the basic medium, this causes an increase in dislocation density (δ), which is inversely proportional to the grain size/crystallite size calculated using Scherrer's formula use for ZnO Nano-swirlings (ZNsw). The elevated ‘δ’ readings are calculated for approximated sizes around 16 and 48 nm. It is noticeable that as crystallite sizes decreases, dislocation density increases. In comparison, the average crystallite size values obtained for W–H analysis varied significantly, which was due to differences in particle distribution averaging. The crystal size determined by the Williamson-Hall equation does not agree with that determined by Scherrer's equation (having crystallite sizes of ∼16 to ∼48 nm), implying that Scherrer's formula fit well for the quantification of ZNsw crystals. In basic medium, there was variation and an increase in lattice strain. Thus, the variance in strain could well be due to changes in particle size/structure^30^.

**Figure 2:**
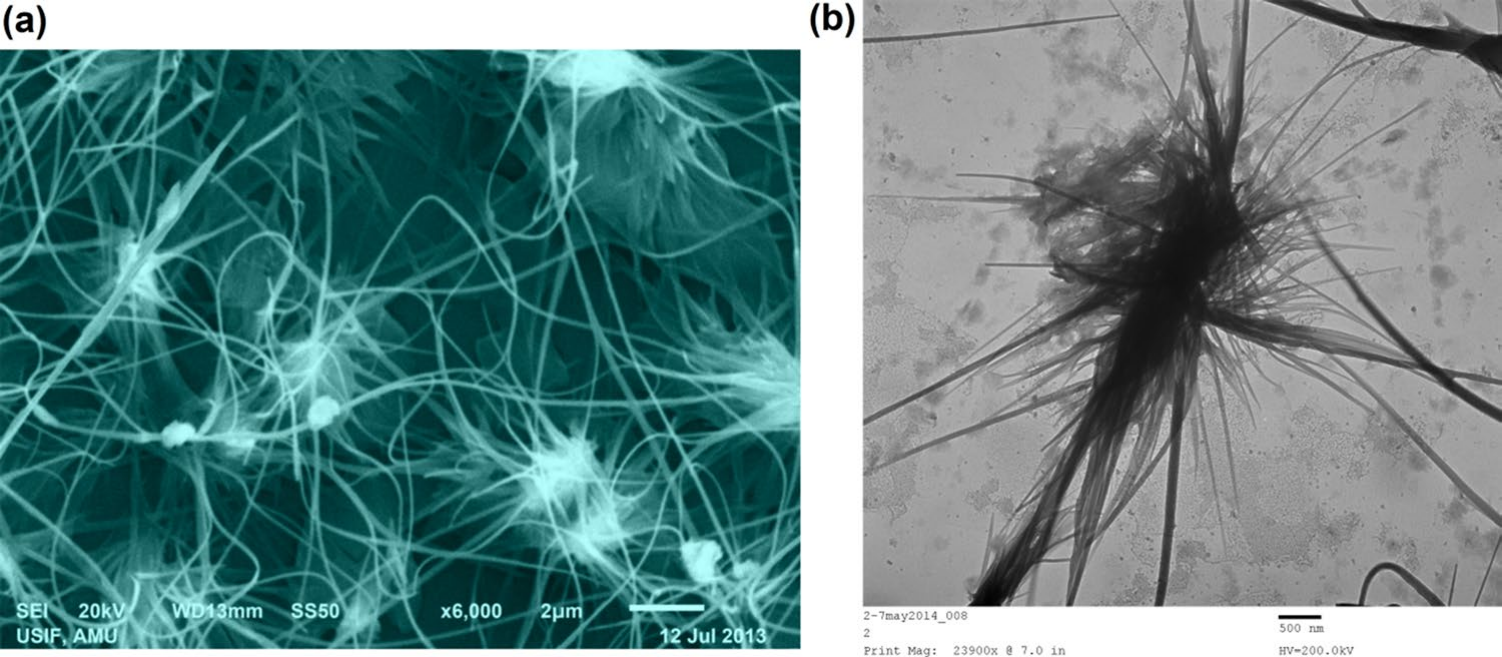
(**a**) SEM image and (**b**) TEM image of as synthesized ZNsw via sol–gel process.

**Figure 3:**
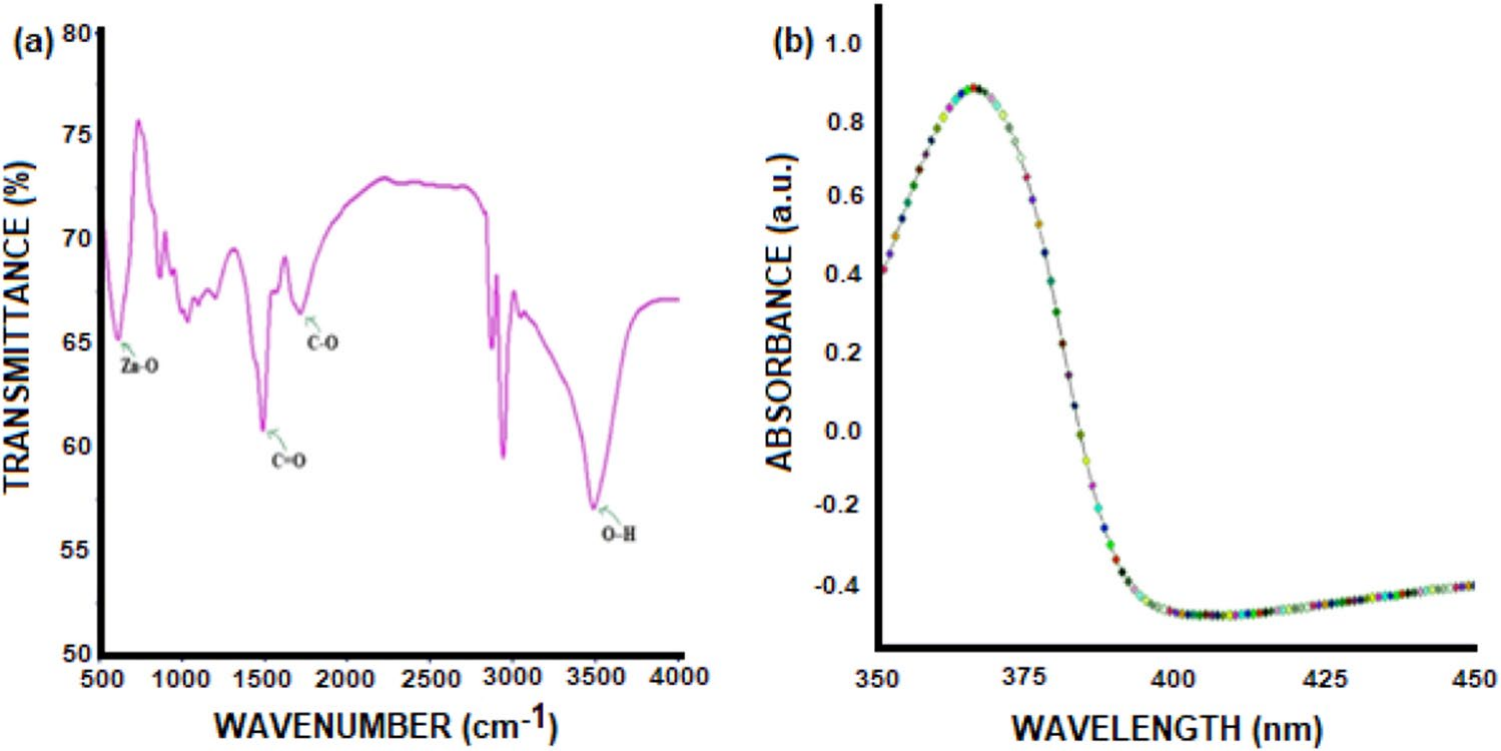
(**a**) FT-IR spectrum and (**b**) UV–Vis spectrum of as synthesized ZNsw via sol–gel process.

The chemical structure and quality of synthesized ZNsw samples are evaluated by X-ray dispersive energy spectrometry (EDX) as seen in Fig. 1b. Only zinc and oxygen were found in the synthesized ZNsw samples, according to the EDX spectrum. In comparison to the Zn and O peaks, there are few extra peaks, presumably due to the existence of a substrate on which the ZNsw sample was held throughout instrumentation. SEM results as well as micrographs have considered the surface morphology of synthesized ZNsw and are shown in Fig. 2a. This indicates the specimen of ZNsw formulated at >> 1900 rpm, contains a huge network of ZNsw, containing a significant quantity of thread-shaped web like structure having a fair amount of the concentration in these webs as observed in Fig. 2a cluster. This also marked the extensive length when compared with the crystal diameters, so their mean aspect ratios would also ≈ length of the thread curling. It is apparent from the figure ZNsw formed are very prominent and are guided towards the edges as for simple 1D thread syntheses, such as ZNsw and the same growth pattern previously mentioned^31^. Furthermore, TEM analyzed the characteristics of the surface morphology. The details of surface morphology as well as the confirmation of the fabricated ZNsw in the range of nano-sizes were confirmed by TEM as depicted in Fig. 2b, Here the TEM results fully matched with SEM observation. Fourier Transform Infrared Spectroscopy (FT-IR) was used to characterize the chemical characteristics. For this reason, the study is carried out at ambient condition between 400 and 4000 cm^−1^ and shown as FT-IR spectra in Fig. 3a. The 589 cm^−1^ stretching bands correspond to ZnO's characteristic absorption band^32^. O–H (around 3452 cm^−1^); C–O (around 1449 cm^−1^), and C=O (around 1549 cm^−1^) were the other signature vibration modes. The other distinctive vibration modes were O–H (about 3452 cm^−1^), C–O (near 1449 cm^−1^), and C=O (about 1549 cm^−1^), which was unexceptional attributable when measuring FT-IR in the air^33^. The synthesized ZNswis further distinguished by the spectrum of UV–vis absorption to illustrate the architectural and optical characteristics as shown in Fig. 3b. A powerful short absorption-band is observed around 366 nm, which is free of all other peaks. The peak observed is a substantial and characteristic peak for the absorption of pure hexagonal ZnO wurtzite^34,35^.

### Growth of ZNsw

By observing the reaction engaged in the synthesis method, the development of ZNsw can be easily understood. The CTAB solution is followed with the incremental addition of zinc acetate dihydrate solvent with continuous agitation in a standard process condition. It was important to observe that there have been no immediate improvements among the reactants. But then, the zinc acetate dihydrate disintegrates to create the zinc cation (Zn^2+^), which then combines with the hydroxyl ion to form the zinc hydroxide^36^. The reactions are as follows:

$${\text{Zn}}\left( {{\text{CH}}_{3} {\text{COO}}} \right) \cdot 2{\text{H}}_{2} {\text{O}} + {\text{DD Water }} \to {\text{Zinc cation + }} {\text{CH}}_{3} {\text{COO}}^{2 - }$$

$$\left( {{\text{CH}}_{2} } \right)_{6} {\text{N}}_{6} + {\text{DD}}\;{\text{Water}} \to 6{\text{HCHO}} + 4{\text{NH}}_{3}$$

$${\text{NH}}_{3} + {\text{DD}}\;{\text{Water}} \to {\text{NH}}_{4}^{ + } + {\text{Hydroxyl}}\;{\text{ion}}$$

$${\text{Zinc}}\;{\text{cation }} + {\text{Hydroxyl}}\;{\text{ion}} \to {\text{ Zn}}\left( {{\text{OH}}} \right)_{2}$$

In the beginning, as the resulting solution's pH increased with the incremental addition of NaOH solution, the hydroxyl ion; OH^−^ generated and zinc hydroxide; Zn(OH)_2_ was subsequently reacted to give Zn(OH)_4_^2−^ as:

$${\text{Zn}}\left( {{\text{OH}}} \right)_{2} + {\text{Hydroxyl}}\;{\text{ions }} \to {\text{Zn}}\left( {{\text{OH}}} \right)_{4}^{2 - }$$

Zn(OH)_4_^2−^ was highly pH as well as temperature dependent step. To create a transparent solution without rising temperature, the nucleation of the source compounds was regulated by the basicity of the solution (Zinc cation / Hydroxyl ion < 1:10). The pH of the appearing solution has been demonstrated to have a substantial impact on nanomaterial structure determination. Highly controlled ZNsw were obtained under intermediate concentration (1:6 < Zinc cation/Hydroxyl ion < 1:7)^37^. Many people have reported successfully in recent years that surfactants play a vital role in ZnO nano-synthesis. The “oil–water” medium, that uses prototype surfactants, serves as effective nano-reactors or so-called distinct micelles where augmentation of crystals appears. However, the template function of surfactant is not always persuasive, as very broad exceptions are formed for anisotropic assemblies. As the reactions were performed in high pH, the solution was abundant in hydroxyl ions. Due to the significant size variation among negatively charged Zn(OH)_4_^2−^; OH^−^ and CTAB assembled; the freshly generated small crystals were predominantly covered with substantial amounts of hydroxyl ions, CTAB also enables the movement of growth-units of Zn(OH)_4_^2−^, that come together to form a structure that resembles an individual rod-shaped assembly into a morphology of ZNsw. The Zn(OH)_4_^2−^ ions segregate as the reaction proceeds and ages to form ZnO nuclei as follows:

$${\text{Zn}}\left( {{\text{OH}}} \right)_{{4}}^{2 - } \to {\text{ZNsw}}^{^{\prime}} \;{\text{crystals}}\;{\text{ nuclei}} + {\text{Hydroxyl}}\;{\text{ ions}}$$

By preferential c-axis [0 0 0 1] directed growth, these ZnO nuclei typically transforms into nanorods, as the growth rate towards the [0 0 0 1] is more in comparison to other facets^38^. It was significant to note that both ends of the rods were glued to their ends and assembled into groups of hexagonal-shaped multiple rods that develop curlings by growth towards polar [0 0 0 1] and –[0 0 0 1] surfaces at the same normal rate growth as stated in the literature^39,40^. The length of these nano-rods varies from 100 nm to micro-metres and finally, multiple nano-curlings growth from nuclei centre is concealed. Highly crystalline nanorods grew radially from the same core as ZNsw Nano rods, as shown in Fig. 2. Exclusive ZNsw crystals with sizes in the overall range below 100 nm are also shown in Fig. 2. This overall takes place under meticulous agitation condition (of >> 1900 rpm)^41^. On the breakdown of reactants, as well as owing to high agitation conditions, a substantial quantity of energy, temperatures, and pressures is reported to be generated, and this energy is high enough to overcome the energy barriers needed for the growth of ZNsw crystals^42^. Hence the formation of ZNsw crystals is the outcome of the interaction of capping agents, precipitating agents together in a pre-defined agitation condition at particular pH.

### Azo Dye AR183 photocatalytic deterioration in the presence of ZNsw

**Figure 4:**
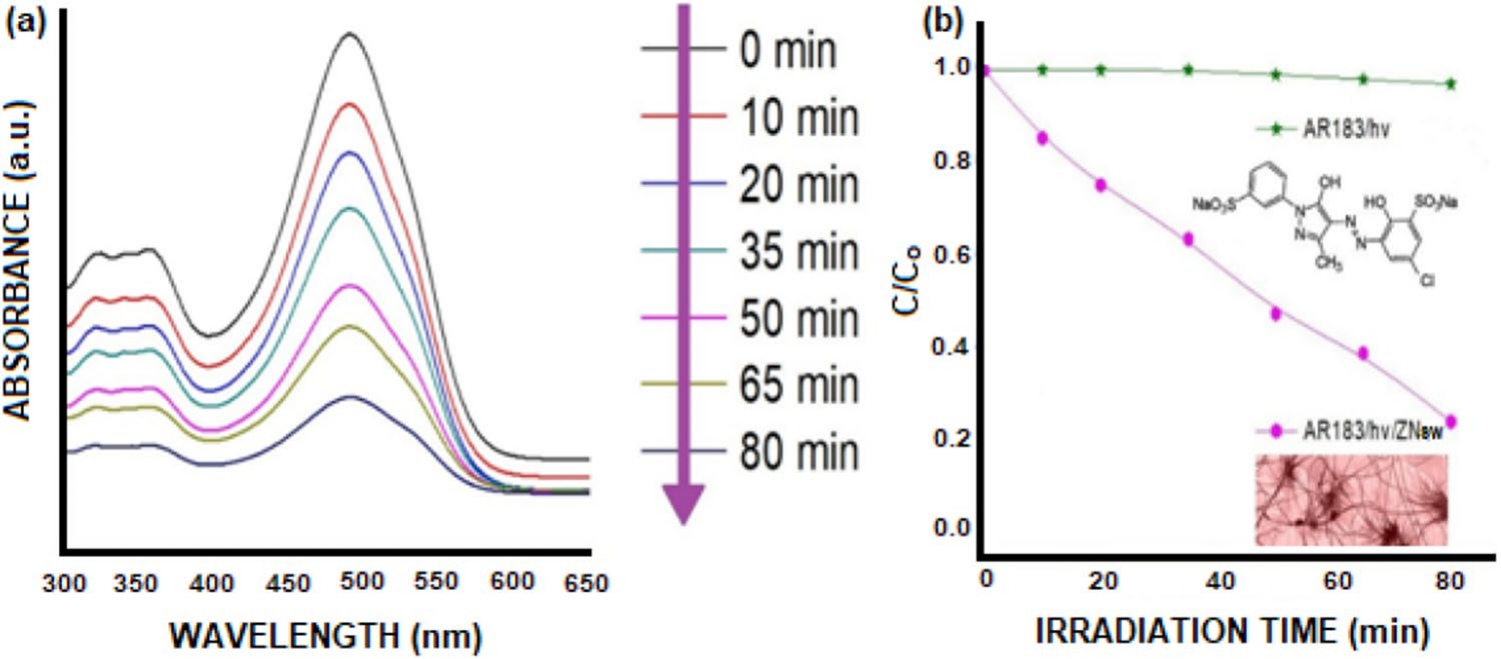
(**a**) Photodegradation of Azo Dye AR183 with ZNsw and (**b**) Change in concentration as a function of time on irradiation of an aqueous solution of Azo Dye AR183 in the absence and presence of ZNsw.

To examine the photocatalytic deterioration of Azo Dye AR183, the synthesized ZNsw were utilized as a photocatalyst. The peak of AR183's UV–visible absorption at 490 nm (λ_max_) is shown in Fig. 4a, which reduced significantly with an increase of time from 0 to 80 min under UV-irradiation. The gradual decline in proportional intensities of absorption can be noticed under UV light, as Azo Dye AR183 is degraded by the produced ZNsw as a photo-catalyst. To study the photo-stability of ZNsw, the irradiation of an Azo Dye AR183 aqueous solution, in the absence and availability of ZNsw, was analyzed by change in their concentrations with respect to the time (shown in Fig. 4b). The extent of deterioration of ZNsw surface is usually determined by the equation as:

$${\text{Degree }}\;{\text{of}}\;{\text{ Deterioration}}\left( \% \right) = \left( {{\text{C}}_{{\text{o}}} - {\text{ C}}/{\text{ C}}_{{\text{o}}} } \right) \times 100$$

**Figure 5:**
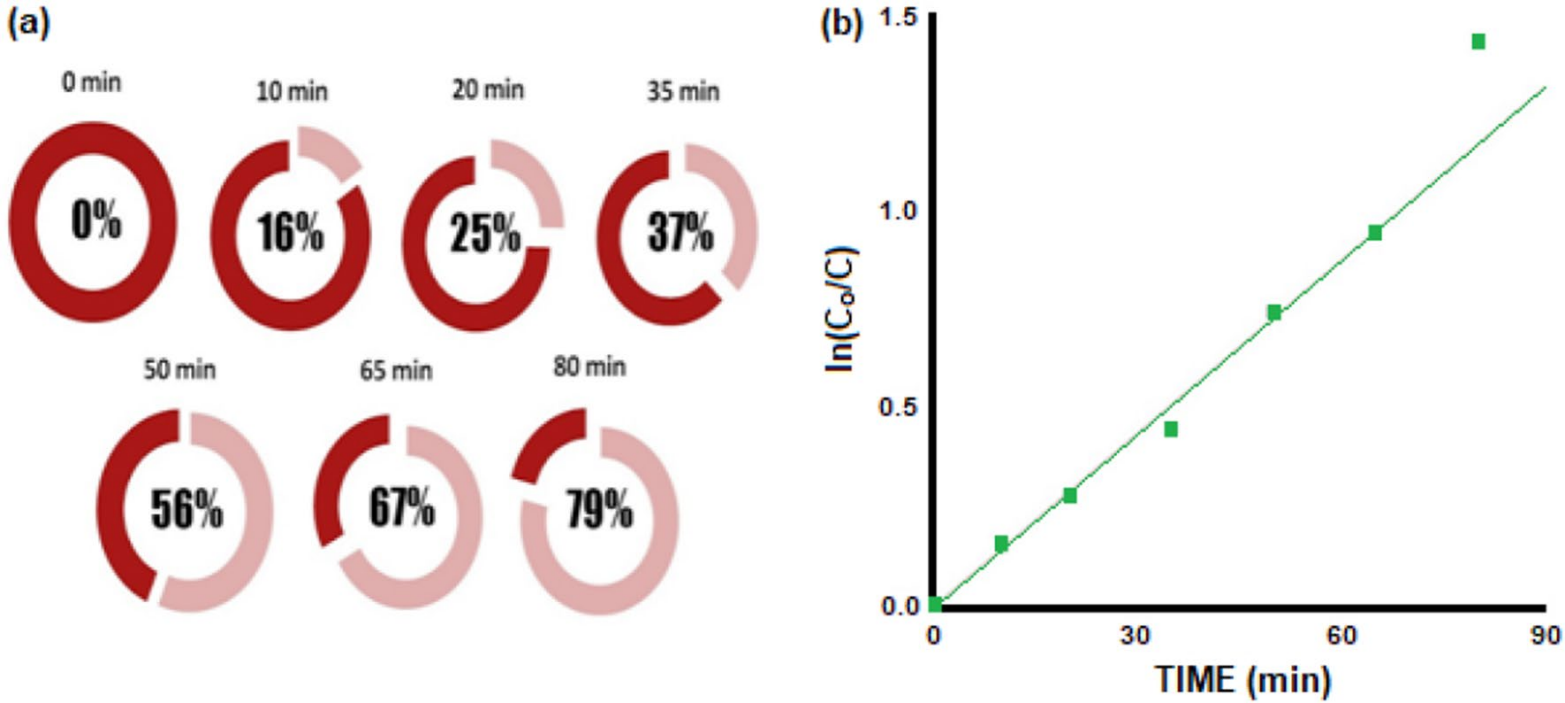
(**a**) Percentage degradation of Azo Dye AR183 with ZNsw versus Irradiation time (min^−1^) and (**b**) Pseudo-First order kinetics based upon Langmuir–Hinshelwood (L–H) kinetic treatment model for Azo Dye AR183 photodegradation in the presence of photocatalyst ZNsw.

This reflects the Azo Dye AR183 concentration C_o_ (at time = 0) and concentration C (at time = t). Figure 4b Illustrate how, in the absence of ZNsw, there was no noticeable deterioration under UV-irradiation, whereas, in the existence of photocatalyst ZNsw, it was important to notice that approximately 79% of the discoloration occurs after 80 min. Figure 5a shows the deterioration percentage of AR183 using ZNsw with irradiation time in minutes. The kinetics associated in the photocatalytic breakdown of Azo Dye AR183 using ZNsw were investigated using the Langmuir–Hinshelwood (L–H) kinetic treatment model^43,44^.

The Fig. 5b depict the graph plotted between in (C_o_/C) versus irradiation time (t). It is based on the L–H kinetic model mentioned as:

$${\text{In}}\left( {{\text{C}}_{{\text{o}}} /{\text{C}}} \right) = {\text{kt}}$$

where *C*_*o*_ Initial AR183 concentration (ppm); *C* AR183 concentration after irradiation time ‘t’; *k* Rate constant (min^−1^); *t* Irradiation time of AR183 (min).

**Figure 6:**
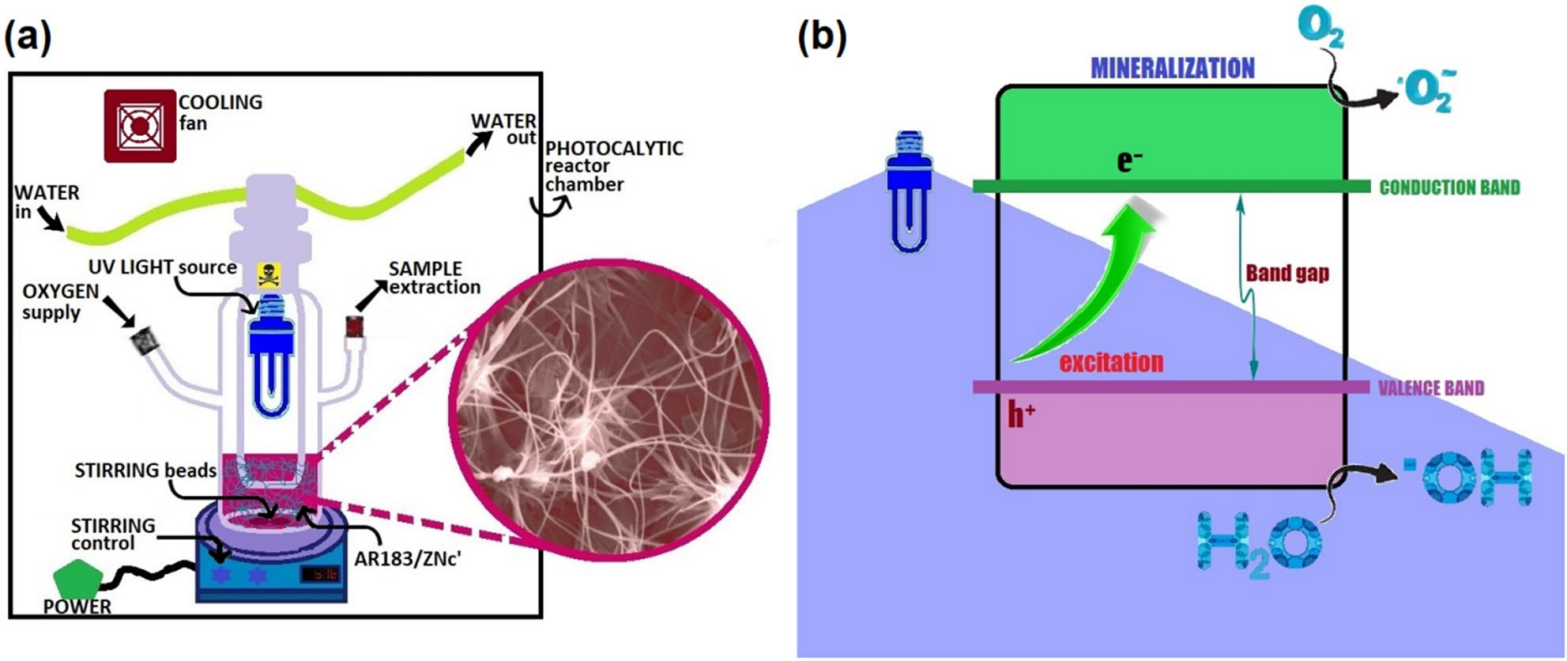
(**a**) UV-Photoreactor with Azo Dye AR183 & photocatalyst ZNsw and (**b**) Schematic representation of photocatalysis mechanism used with Azo Dye AR183.

This graph shows the linear behaviour between In(C_o_/C) versus irradiation time (t). Hence, indicating that the photocatalytic deterioration of Azo Dye AR183 using ZNsw is well-presented by pseudo-first order kinetics based on Langmuir–Hinshelwood (L–H) kinetic treatment model^45^. The value of ‘k’ is calculated from the slope of the graph and is found to be as 1.51 × 10^−2^ min^−1^ and their half-life (t_1/2_) time for the photocatalytic deterioration of Azo Dye AR183 using ZNsw is figured out to be around 45.75 min^46,47^. The process of Azo Dye AR183 deterioration reaction induced by ZNsw under UV light is clarified on the basis of what has been documented in previous studies^13,14^. For the purpose of this study, the photo-reactor used in experimentation is schematically represented in Fig. 6a. The photocatalytic reactions are often referred to as photo-induced reactions because photocatalytic reactions are caused when a photon with a sufficient wavelength (energy equal to or larger than the band gap of the photocatalyst, i.e. hv ≥ Band Gap Energy ≈ 3.3 eV) induces molecular excitation of the photocatalyst. As shown in Fig. 6b molecule excitation contributes to electron promotion from the valence band level (vbl) to the conduction band level (cbl) and hole creation inside the valence band level^48^. Thus, the photocatalytic deterioration occurs through a sequence of chemical reactions; the initiation stage of the reaction is the creation of an electron–hole

$${\text{ZNsw}} + {\text{ hv}} \to \, {\text{e}}^{ - }_{{({\text{cbl}})}} + \, {\text{ h}}^{ + }_{{({\text{vbl}})}}$$

This result in the use of a hole for the oxidation reaction and an electron to produce superoxide anions and hydrogen peroxide (H_2_O_2_) from oxygen for the reduction process, leading to the formation of ^**•**^OH radicals as:

$$\left\{ {{\text{H}}_{2} {\text{O}} + {\text{h}}^{ + } } \right\}_{{({\text{cbl}})}} + \left\{ {{\text{O}}_{2} + {\text{ e}}^{ - } } \right\}_{{({\text{vbl}})}} \to \left\{ {{\text{H}}^{ + } + {\text{OH}}^{ \cdot } } \right\} + \left\{ {{\text{O}}_{2}^{ \cdot - } } \right\} \to {\text{HOO}}^{ \cdot}$$

$${\text{HOO}}^{ \cdot } + {\text{e}}^{ - } /{\text{h}}^{ + } \mathop{\longrightarrow}\limits_{{{\text{h}}^{ + } }}^{{{\text{e}}^{ - } }}{\text{H}}_{2} {\text{O}}_{2} \mathop{\longrightarrow}\limits^{{{\text{e}}^{ - } }}\;^{ \cdot } {\text{OH}} + {\text{OH}}^{ - }$$

Finally, we may conclude that the processes at the semiconductor surface that caused dye degradation went like this^7^:

$${\text{Azo}}\;{\text{Dye}}\;{\text{AR}}183 + \left\{ { \cdot {\text{ OH}}\;{\text{or}}\;{\text{h}}^{ + } } \right\} \to \to {\text{Intermediates}} \to \to {\text{Mineralized}}\;{\text{Products}}$$

These redox reactions occur on the semiconductor surface. There is a risk of electron–hole pair recombination if the photo-induced electron–hole pair did not split properly, which would reduce the performance of photocatalytic activities. So, any attempt to avoid electron and electron–hole recombination would help increase photo-degradation performance and to achieve better photocatalytic applications^34,49^. The drop in concentration tracked a pseudo-first-order kinetic, with the constant k equal to 1.51 × 10^−2^ min^−1^. After 80 min of irradiation, massive deterioration rates of 79% were recorded. As presented in figures, the photo-degradation of Azo Dye AR183 with ZNsw was similar^50^.

Photo-degradation has several benefits over previous wastewater treatment systems such as biological treatment, chemical oxidation, activated carbon adsorption, and so on. The effectiveness and oxidation levels of the photocatalytic system are immensely essential for a variety of control parameters managing the photo-degradation of different molecules^51^. A specific orientation of the dye and photocatalyst could be responsible for the improved photo-degradation of Azo Dye AR183^52–54^.

### Azo Dye AR183 photocatalytic mineralization in the presence of ZNsw

**Figure 7:**
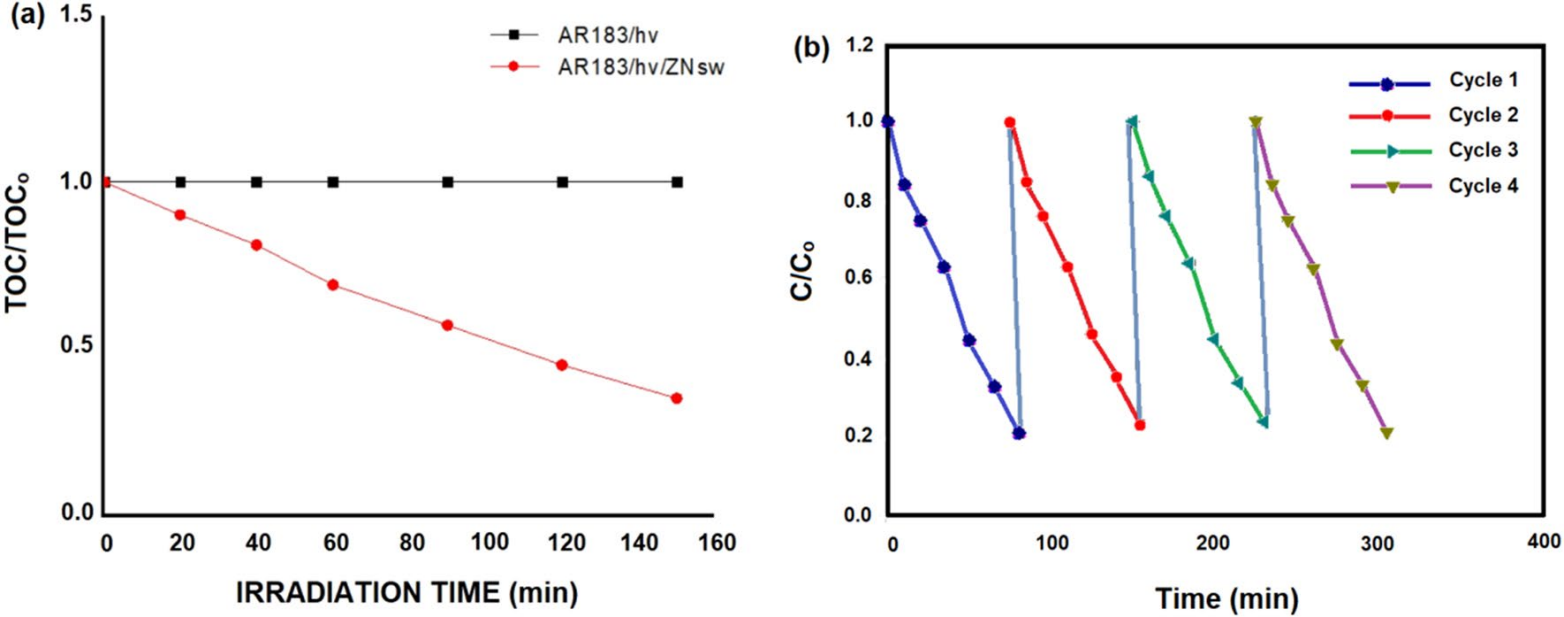
(**a**) Azo Dye AR183 photocatalytic mineralization in the presence of ZNsw and (**b**) Reusability of ZNsw in the photo-degradation of Azo Dye AR183.

Photocatalytic mineralization of Azo Dye AR183 was studied in the presence as well as in the absence of ZNsw with constant supply of oxygen. Figure 7a shows the depletion in Total organic carbon (TOC) content as a function of time on irradiation of an aqueous suspension of AR183 in the presence of ZNsw. It could be seen from the figure that degradation was almost negligible when the irradiation was carried out in the absence of photocatalyst. However, 79% degradation was observed in 80 min when irradiation was carried out in the presence of ZNsw.

In addition, the synthesiszed ZNsw was dissolved in distilled water and was kept on shaker for 6 h. After centrifugation, the sample was analysed by Atomic absorption spectrometer (AAS-PerkinElmer An Analyst 400; Waltham, MA, USA). It was observed that there was no detectable amount of free Zn^2+^ ions in the results.

### ZNsw reusability method

The reusability of ZNsw in the photo-degradation of Azo Dye AR183 was further investigated. AR183 photocatalytic deterioration resulted in a solution that was condensed, cleaned, and dried. Under comparable conditions, the dried catalyst samples were re-used for Azo Dye AR183 degradation. The filtrate was tested for ZNsw ion loss to solutions, and AR183 dissolution was found to be negligible. The photo-catalyst ZNsw can be reused without any special procedures, and the photocatalytic activity of the reused catalyst was reported to be the similar as that of the initial catalyst after four batch reactions. The operation and decline of ZNsw ions became more apparent after the fourth stage of reuse. The photodegradation of AR-183 during four consecutive cycles is depicted in Fig. 7b. After 80 min of reaction time, the relative decolorization utilizing ZNsw for the 4-cycling reuse was 79.0%, 78.3%, 77.8% and 77.1% respectively. The explanation for this drop could be due to ZNsw runoff during washing and filtering, which has a constant decolonization rate.

### Antimycotic activity in the presence of ZNsw

The inhibition in growth of plant pathogens was found to be 80.55% and 56.66% after 6 days of inoculation of *A. alternata* and *R. solani* against ZNsw, respectively. The radial growth of *A.alternata*was 1.75 cm against 9 cm in control, inhibiting around 80.55%. Meanwhile the radial growth of *R. solani* was 3.9 cm against 9 cm in control which inhibited 56.66%. After 6 days of inoculation, the radial development of *A. alternata* was dark green and low dense in appearance, suggesting that plant pathogens had been inhibited by ZNsw. Similarly, *R. solani *produced white dense mycelial growth after 6 days of inoculation, showing the efficacy of ZNsw against the plant pathogens. However, ZNsw was more effective against *A. alternata* as compared against *R. solani* (shown in Table 1). These two pathogens pose significant threats to the production of economic crops. ZNsw has proved to be an excellentantagonistic activity. Therefore, the use of ZNsw can be likely to serve as an effective fungicide to kill the plant fungal pathogens in agricultural practices in addition to treating the polluted water. The ZNsw is environmentally acceptable and could efficiently play a significant role in reducing fungal pathogenicity in agricultural production systems.

**Table 1:** Antifungal activity of ZnO Nano-swirlings at different stirring conditions (after 6 days of Inoculation).

Sample	Radial growth (cm) average of triplicate	% Inhibition ($= \frac{{{\text{C}} - {\text{T}}}}{C} \times 100$)	Control (cm)	Color	Treatment
*Alternaria alternata*					
ZNsw	1.75	80.55%	9	Dark Green & Low dense	
*Rhizoctonia solani*					
ZNsw	3.9	56.66%	9	White & Dense mycelial growth	

*ZNsw* ZnO Nano-swirlings; *C* Diameter of control colony; *T* Diameter of treated colony.

## Conclusion

The production and analysis of ZNsw using the sol–gel technique under a predetermined agitation condition (of >> 1900 rpm) have been documented, and the effects of agitation conditions on crystallite size, morphologies, and optical design have been analysed. The SEM and TEM results show that zinc oxide formed a large cluster of adequate ZNsw with a significant amount of folded long thread-lengths. The photocatalytic operation was calculated effectively by specifically following the photo-degradation of Azo Dye AR183 using the photo-catalyst, ZNsw. It was interesting to observe that about 79% of discolouration takes place in just 80 min of irradiation in the presence of ZNsw whereas there was no substantial degradation seen under the same conditions without ZNsw, indicating that the synthesized ZNsw works as excellent photocatalyst. Additionally, the ZNsw antagonistic efficacy for plant pathogens was determined concerning the antimycotic action. The per cent growth inhibition was 80.55% and 56.66% against *A. alternata* and *R. solani,* respectively. The ZNsw is environmentally friendly and has the potential to play an important impact in mitigating fungal pathogenicity in agricultural production systems.

## Experimental details

### Materials

Zinc acetate dihydrate and sodium hydroxide purchased from E. Merck Limited (India). Cetyl trimethyl ammonium bromide (CTAB) was purchased from Loba (India). Merck KGaA (Germany) provided the Azo Dye AR183. They were of analytical accuracy without any further processing and H_2_O undergone double-distillation (DD) was used during the synthesis.

### Synthesis of ZNsw

Approximately 0.2 g of CTAB was first applied to H_2_O undergone DD water, in a flat bottom flask, after continuous agitation and mild heating for rapid production of ZNsw. Later, 5% of zinc acetate dihydrate (21.94 gm) solution in 0.1 L was introduced into the reaction mixture. Slow but steady dropwise addition of precipitating agent, sodium hydroxide solution at predefined agitation condition (of >> 1900 rpm) before solution of colloidal property becomes milky white. After retention in the desiccators, the setup was cooled as well as dried and then washed with purified water accompanied by alcoholic methanolsolution. Without any specialized procedure, the entire synthesis was carried out.

### Characterization of ZNsw

ZNsw structural architecture as analyzed by SEM from JEOL JSM-6510 LV fitted with Oxford EDS system and TEM from JEM-2100F, as prepared. For scanning electron microscopy, the setup gives an output of the 200 kV with the scale size of the probe below 5 × 10^−1^ nm. ZNsw Powder XRD from Bruker AXS. D8 Advance with Cu-radiation Kα; λ = 1.54178 Å with a scanning speed of 4° min^−1^ between the range of 30° to 70° was used to examine the crystal phase and crystalline nature of the desired sample. The chemical properties were determined using the FT-IR from Thermo Scientific Nicolet is-10 (Massachusetts, USA), produced by electro-magnetic radiation absorption between 400 and 4000 cm^−1^ frequency range. The optical characteristics of produced ZNsw were investigated using diffuse UV reflection spectroscopy and UV–vis absorption spectroscopy at room temperature, by using Lambda 850 UV/vis spectro-photometer (Perkin-Elmer Life and Analytical Sciences, CT, USA) in the range of 300–450 nm operating at a resolution of 1 nm, in the quartz cuvette of 1 cm path length. The DD water was used as a reference material for background correction.

### Photocatalytic activity of ZNsw

By effectively tracking the dye removal process under UV irradiation, the photocatalytic process of synthesized ZNsw has been evaluated. The irradiation period in the presence of ZNsw under UV light resulted in the photocatalytic degradation of Azo Dye AR183. A photocatalytic well-immersed reactor finished of pyrex glass, an intermediate pressure mercury lamp of 125 W (1.74 to 1.78 mWcm^−2^), fitted with a magnetic stirring rod, a water movement sheath, and an ambient O_2_ supply nozzle. A pyrex glass sheath with water circulation reduces UV-radiations with short wavelengths. To make a 10 ppm sample, a sufficient quantity of AR183 was enforced to the purified water. To achieve a balance, the aqueous dye solution of 0.25L was inserted into the photo-bioreactor, and roughly 2 gmL^−1^ ZNsw was utilized, and then the resulting aqueous mixture was stirred in the dark for at least 0.25 h with ambient O_2_, hence maintaining the system equilibrium. The aqueous mixture of stable dye will be left vulnerable to ultraviolet light radiation. The sample was selected at a normal time interval from the photo-bioreactor, which was then centrifuged to clean the photo-catalyst, and then a UV–visible absorption spectrophotometer was used to detect the absorption of the damaged dye solution.

### Antimycotic activity of ZNsw

The two phytopathogenic microbial fungus *R. solani*and *A. alternata* were acquired first from Microbial Type culture collection (IMTECH, Chandigarh, India). The two microbial pathogens were cultured at 27 °C in the dark on PDA (Potatoe-Dextrose Agar). Antifungal tests were carried out using a modified agar dilution technique. ZNsw were treated with PDA medium at a 1.0 (mM) concentration, with ZNsw free PDA serving as a control. After the PDA medium had hardened, the test along with control media were placed into sterilized petri-dishes (with a diameter of 9 cm), and the inoculation fungalstrains were done. As from the fungal cultures samples with 7 days’ older edges, a disc of fungal-mycelial matter (1.4 cm) was extracted. The disc was put in the centre of each Petri dish and then the parafilm sealed to avoid dehydration. At 27 °C, the Petri dishes were incubated (treated/untreated). By taking measurement of the fungal colonies diameter and then using the following calculation method, the efficiency of as-prepared composites was assessed after 12 days, in terms of percent suppression of fungalcolonies. The test procedures were done in triplicate, and the findings were presented in centimetres.

$$\% \;{\text{inhibition}}\;{ = }\;\frac{{{\text{Diameter}}\;{\text{of}}\;{\text{control}}\;{\text{colony}} - {\text{Diameter}}\;{\text{of}}\;{\text{treated}}\;{\text{colony}}}}{{{\text{Diameter}}\;{\text{of}}\;{\text{control}}\;{\text{colony}}}}$$

## Data Availability

All data generated or analysed during this study are included in this article.
